# RNA Degradation in *Staphylococcus aureus*: Diversity of Ribonucleases and Their Impact

**DOI:** 10.1155/2015/395753

**Published:** 2015-04-21

**Authors:** Rémy A. Bonnin, Philippe Bouloc

**Affiliations:** Institute for Integrative Biology of the Cell (I2BC), CEA, CNRS, Université Paris-Sud, Université Paris-Saclay, 91400 Orsay, France

## Abstract

The regulation of RNA decay is now widely recognized as having a central role in bacterial adaption to environmental stress. Here we present an overview on the diversity of ribonucleases (RNases) and their impact at the posttranscriptional level in the human pathogen *Staphylococcus aureus*. RNases in prokaryotes have been mainly studied in the two model organisms *Escherichia coli* and *Bacillus subtilis*. Based on identified RNases in these two models, putative orthologs have been identified in *S. aureus*. The main staphylococcal RNases involved in the processing and degradation of the bulk RNA are (i) endonucleases RNase III and RNase Y and (ii) exonucleases RNase J1/J2 and PNPase, having 5′ to 3′ and 3′ to 5′ activities, respectively. The diversity and potential roles of each RNase and of Hfq and RppH are discussed in the context of recent studies, some of which are based on next-generation sequencing technology.

## 1. Introduction

*Staphylococcus aureus* is a main source of hospital-acquired infections causing pneumonia, endocarditis, osteomyelitis, soft-tissue, and skin infections [1].* S. aureus* also causes serious nail infections (paronychia) and is a common cause of food poisoning due to the production of enterotoxin [2]. A main problem concerning* S. aureus* infections is its ability to become resistant to multiple antibiotics including *β*-lactams (MRSA) and glycopeptides and also to more recent molecules such as linezolid and daptomycin [1, 3, 4]. In the mid-1990s, the emergence of community-acquired antibiotic-resistant staphylococcal infections in individuals with no identified risk factors raised new concerns [5]. The underlying factors of* S. aureus* pathogenicity relate to the coordinated expression of numerous virulence factors. The combined risks of disease and diminishing efficacy of antibiotic treatments have incited the scientific community to investigate staphylococcal transcriptional and posttranscriptional regulation in detail.

**Figure 1:**
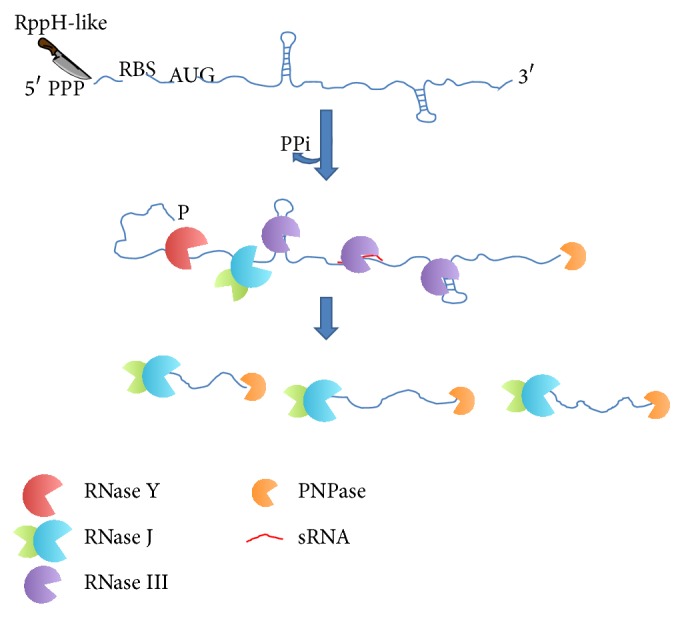
RNA decay in* S. aureus*. The proposed scheme of RNA decay is based on* B. subtilis* and* S. aureus* data. The first degradation step is likely initiated by pyrophosphate removal from 5′ triphosphorylated ends of primary transcripts. This step is catalyzed by RppH-like enzymes and is followed by an RNase Y-dependent endonucleolytic cleavage. RNAs with 5′ monophosphate ends are degraded by the bifunctional enzyme RNase J made of RNases J1 and J2. PNPase degrades RNAs from their 3′ end. Oligonucleotides are then likely degraded into nucleotides by an oligoribonuclease.

RNA steady-state maintenance is the result of synthesis and degradation of transcripts. In contrast to eukaryotes, bacterial mRNAs are usually short-lived with a half-life ranging from a few seconds to over one hour. Ribonuclease (RNase) activities contribute to RNA processing or degradation. RNA processing is a cleavage leading to functional transcripts, while RNA degradation results in RNAs transformed into oligonucleotides and nucleotides. Transcriptional and post-transcriptional regulatory pathways control protein production and contribute to homeostasis and adaptation to environmental stress. In bacteria, the first step of RNA decay is generally thought to involve removal of the RNA 5′-end pyrophosphate. It is followed by an endonucleolytic cleavage allowing exonucleolytic degradation. In* Escherichia coli*, exonuclease activity is solely 3′ to 5′ while in* Bacillus subtilis*, 5′ to 3′ exonuclease activity was uncovered. Extensive studies on RNA processing and degradation reveal that while several RNases are present in other species of their respective taxons (e.g., RNase III, PNPase, RNase R, RNase P, and RNase Z) (Figure 1 and Table 1), RNase E, while essential in* E. coli*, is absent in* B. subtilis*. Instead, RNase J1, RNase J2, and RNase Y are present in* B. subtilis* and for some aspects are functional homologs of RNase E.

Since RNases were primarily studied in the Proteobacteria* E. coli* and the Firmicutes* B. subtilis*, results obtained for these species will be presented to discuss the recent knowledge on RNA decay in* S. aureus*.

## 2. Main RNases Identified in* S. aureus*

### 2.1. The Double-Strand RNA-Specific Endonuclease RNase III

RNase III is a double-strand (ds) specific RNase discovered in* E. coli* extracts more than forty years ago [6]. Its activity is divalent cation-dependent and is inhibited* in vitro* by metal chelators [6]. RNase III-family enzymes show a large diversity in terms of primary protein structure, ranging from the* B. subtilis* Mini-III RNase (143 amino acids; Uniprot O31418) to the large* Homo sapiens* Dicer1 protein (1,922 amino acids; Uniprot Q9UPY3). However, all family members possess a common RIIID-like domain that includes a nine-residue signature motif [7].

Through its ds-RNA specificity, RNase III is a key player in various cell processes. These roles include the maturation of ribosomal RNAs (rRNA) by cleaving stem-loops inside the primary rRNAs [8–10] and mRNA processing including its own mRNA by cleaving a stem-loop involved in a feedback autoregulation [11]. One of the first discovered roles of RNase III was its implication in the lifestyle of temperate bacteriophage *λ*. RNase III cleaves a stem-loop in the 5′UTR region of the N gene transcript, thus releasing the Shine Dalgarno (SD) sequence and permitting recruitment of ribosomes [12]. Due to its ds-specific RNase activity, RNase III is also involved in cleaving small regulatory RNA (sRNA)/mRNA duplexes [13, 14]. Recent studies in* Streptococcus pyogenes* show that RNase III acts in concert with the CRISPR Csn1 protein to mature CRISPR RNAs (crRNA), resulting in prophage silencing [15].

In* B. subtilis*, amounts of 470 transcripts, representing 11% of total transcripts, were shown to be altered by RNase III depletion [13]. However, RNase III essentiality was due neither to its global role on bulk RNA level nor to rRNA maturation, but to its role in the elimination of toxins encoded by type I toxin/antitoxin (TA) systems. The deletion of* txpA/ratA* and* yonT/as-yonT* TAs was sufficient to suppress the RNase III essentiality [16].

**Figure 2:**
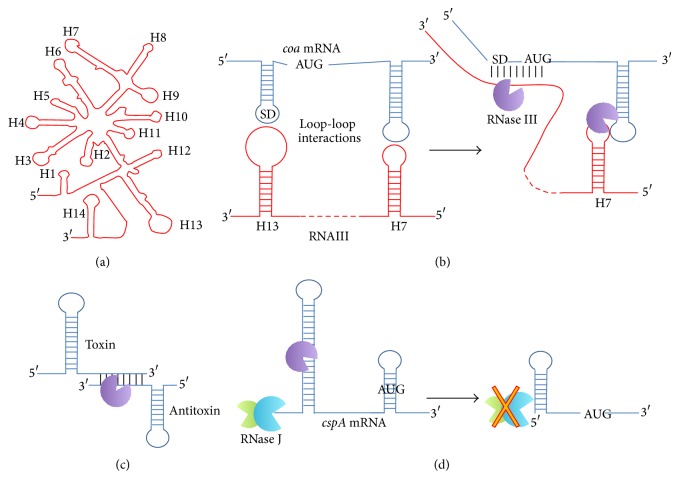
Examples of RNase III functions (a) Schematic view of* S. aureus* RNAIII structure. RNAIII is involved in the regulation of virulence genes by base-pairing with specific mRNAs [57]. (b) The region of* coa* mRNA (encoding coagulase) close to its Shine-Dalgarno sequence base-pairs with the RNAIII helix H13 and is stabilized by a second interaction involving the RNAIII helixH7. RNase III degrades the* coa* mRNA-RNAIII duplex, both in the SD region and within the loop-loop interaction region. (c) RNase III degrades ds-RNAs including sense-antisense RNA duplexes as exemplified by type I toxin-antitoxin systems [16]. (d) Cleavage inside a stem-loop can give rise to a more stable mRNA, as demonstrated for the cold shock protein A* cspA* mRNA. Cleavage of the stem-loop releases the translation start codon and a new stem-loop protects the 5′ end from RNase J-mediated degradation [24].

RNase III is the most studied* S. aureus* RNase; its role was mainly determined through the characterization of virulence genes regulated by the* agr* system [17–20]. RNAIII, a 514 nucleotide regulatory RNA which base-pairs with numerous targets, is the* agr* system effector (Figure 2(a)) [20, 21]. The staphylococcal protein A, encoded by the* spa* gene, inhibits phagocytic engulfment; its mRNA is RNAIII targets. The regulation of* spa* involves the formation of an RNAIII-*spa* mRNA duplex that is then degraded by RNase III [18]. Duplex formation is sufficient to prevent translation of* spa* mRNA;* spa* mRNA degradation by RNase III contributes to the irreversibility of the process. Other examples where mRNA-RNAIII duplex formation leads to a translational arrest and consequent mRNA degradation include (i)* rot* mRNA (encoding a regulator of toxins) through imperfect base pairings involving two loop-loop interactions and of (ii)* coa* mRNA (encoding the staphylococcal coagulase) via the binding of two distant regions of* coa* mRNA (Figure 2(b)) [17, 22]. Toeprinting and RNase cleavage assays demonstrated that RNase III cleaves at the bottom of a stem loop and also inside loop-loop interactions (Figure 2(b)).

Two recent studies gave novel insights at a genome scale on the function of the staphylococcal RNase III [23, 24]. A first approach was based on sequencing of cDNA libraries obtained by coimmunoprecipitation assays with either wild-type RNase III or catalytically inactive but binding-efficient RNase III [24, 25]. These experiments elucidated the roles of RNase III in different cellular processes including (i) rRNA and tRNA processing, (ii) RNase III autoregulation by self-cleavage, and (iii) processing/cleavage of mRNAs and mRNA-sRNA duplexes [24]. Similar roles have been reported in other bacteria [7, 8]. Interestingly, RNase III processes* cspA* mRNA, encoding the cold shock protein CspA. The first step is a cleavage within a long hairpin in the* cspA* mRNA 5′UTR (Figure 2(c)). As a consequence, the mRNA 5′UTR is shortened giving rise to a more stable transcript and rendering the SD sequence accessible for a higher rate of translation. This case exemplified the role of RNase III in stimulating translation efficiency as was demonstrated for the N gene in phage *λ* [7, 12]. In addition to mRNA targets, 58 noncoding RNAs (ncRNAs) were coimmunoprecipitated with RNase III [24]. The use of a catalytically inactive RNase III allows capturing of ds-RNAs, including sRNAs base-paired to mRNAs, so that sRNA targets can be identified at a genome scale.

A second study focusing on the role of RNase III at a genome scale was performed using a comparative transcriptomic analysis of wild-type and RNase III deficient (Δ*rnc*) strains [23]. The authors sequenced cDNA of both long and short (<50 nt) transcripts. A collection of short transcripts covering more than 75% of all mRNAs throughout the* S. aureus* genome was identified. In the absence of RNase III, an accumulation of antisense transcripts and a decrease of short transcripts were observed, suggesting that RNase III likely eliminates a basal level of pervasive transcription [23]. To assess whether this pervasive transcription is common to different bacteria, sequencing of short RNAs was performed for* B. subtilis*,* Enterococcus faecalis*,* Listeria monocytogenes*, and* Salmonella enterica*. A correlation between the absence of RNase III and an increase of short transcripts was observed in all the tested Gram-positive bacteria, but not in the sole Gram-negative species tested (*S. enterica*) [23]. It will be interesting to test whether pervasive transcription is mainly associated with Gram-positive bacteria. Modulation of pervasive transcription by RNase III might have two physiological roles. First, interactions between antisense and sense transcripts could be fine-tuned* via* RNase III, which consequently could control cellular protein levels. Second, RNase III could also eliminate transcriptional noise.

An RNase III paralog, named mini-III due to its small size (143 amino acids in* B. subtilis*), has been described in low GC content Gram-positive bacteria. Mini-III plays a role in the maturation of 23S rRNA in* B. subtilis* [26–28]. However, it can be replaced by the combined activity of RNase J1, RNase PH, and YhaM [29]. A mini-III ortholog is present in* S. aureus* but to date has not been characterized (Table 1).

### 2.2. The Endonuclease RNase Y

The endonuclease RNase Y of* B. subtilis* (encoded by* rny*, formerly* ymdA*) was identified as an RNase that cleaves single-stranded A- or AU-rich sequences [30]. It cleaves SAM-dependent riboswitches, including the* yitJ* riboswitch, but only in the presence of SAM, which contributes to forming a terminator structure. The initial rate of 5′ monophosphorylated RNA degradation is faster than for 5′ triphosphorylated RNAs. However, after prolonged* in vitro* incubation, the same amount of* yitJ* cleaved product was observed even for 5′ end triphosphorylation [30]. These results indicate that RNase Y shows a preference for 5′ monophosphorylated substrates, as observed for RNase E. 5′-dependent and 5′-independent endonuclease activities were observed for RNase Y [31]. Moreover, as RNase Y can bind RNA 5′ ends, it may compete with RNase J for the same substrate (see the following).

RNase Y is involved in the decay of polycistronic* infC-rpmI-rplT* mRNAs encoding the elongation factor IF3 and ribosomal proteins L35 and L20 [32]. This operon is autoregulated by a transcription attenuation mechanism involving L20. When RNase Y is absent, at low L20 concentrations, a longer transcript is stabilized. This transcript expresses L35 and L20, but not IF3. When processed by RNase Y, the transcript is subsequently degraded by RNase J thanks to an entry site for its 5′–3′ exonucleolytic activity [32]. The presence/absence of RNase Y thus influences the level of translation of IF3, L35, and L20. RNase Y* via* its processing activity is also involved in regulation of the* gapA* operon and* bsrG*/SR4 type I toxin/antitoxin system [33, 34].

RNase Y depletion increases the half-life of bulk RNA levels in* B. subtilis* [30]. According to two studies, mRNA abundance is, respectively, increased and decreased for 795 and 309 mRNAs [13] or 550 and 350 mRNAs [35]. The proportion of RNase Y targets in the different studies is similar; however, only 263 candidates were common to both studies maybe due to the use of different depletion mutants. RNase Y depletion has diverse effects, including decreased biofilm formation (due to the stabilization of* sinR* mRNA resulting in the SinR repressor accumulation), modifications in folate and amino acid biosynthesis, extracellular polysaccharide synthesis, and an increase in penicillin-binding protein 2A mRNA stability [35]. Overall, these studies revealed the important role of RNase Y in* B. subtilis* physiology and metabolism.

In* S. aureus*, the* rny* ortholog (aka* cvfA*) was discovered as a regulator of virulence genes using silkworm and mouse infection models [36, 37]. Disruption of* rny* impaired virulence notably by diminishing haemolysin production [36]. RNase Y has a transmembrane domain, an RNA binding domain (KH domain), and a metal-dependent phosphohydrolase domain (HD domain). The integrity of the HD domain is required for the* rny*-dependent phenotypes [38]. As is the case for* B. subtilis*,* rny* is not essential in* S. aureus*, as tested in the NCTC8325 and Newman strains [36, 39]. A recent microarray study in the Newman strain revealed differential expression of about 570 genes between a Δ*rny* mutant and its corresponding isogenic wild-type strain [39]. In a similar experiment, about 520 genes were differentially expressed in the RN4220 background [40]. Many of the affected transcripts do not express proteins. Functional classification of the affected genes indicates that the downregulated genes are mostly involved in pathogenicity or proteolysis whereas the upregulated genes are mainly involved in transport and metabolism [39]. The downregulation of virulence gene expression is linked to processing of the* saePQRS* operon and expression of the two-component system SaeS/SaeR [41]. Different transcripts are produced from the* saePQRS* operon, which differ in stability [42]. RNase Y is the key player for the endonucleolytic cleavage of T1 leading to a more stable T2 transcript and resulting in enhanced* saeRS* translation [39].

### 2.3. The Bifunctional RNase J1/J2

The threonyl-tRNA synthetase leader region from* B. subtilis* expressed in* E. coli* is processed by RNase E, suggesting that an RNase E functional equivalent exists in* B. subtilis* [43]. However, no RNase E homolog is present in the* B. subtilis* genome. Therefore, enzymes having RNase E-like activities (i.e., a role in the maturation of 16S/23S rRNAs and cleavage of the T-box of threonyl-synthetase) were searched in* B. subtilis* leading to the discovery of RNase J1 and RNase J2 (formerly YkqC and YmfA) encoded by* rnjA* and* rnjB*, respectively [44, 45]. As demonstrated by copurification and bacterial double-hybrid techniques, RNase J1 and J2 exhibited strong interactions forming heterodimers and heterotetramers [46]. These enzymes are bifunctional with endonuclease and 5′ to 3′ exonuclease activities, this latter property being until recently considered to be restricted to eukaryotes. However, RNase J2 has poor 5′ to 3′ exonuclease activity compared to the RNase J1 or RNases J1/J2 complex [46]. The exonuclease activity is 5′ monophosphate-end-dependent and single-strand-specific; it is completely inhibited by triphosphorylated ends [47, 48]. The absence of RNase J2 had no effect on bulk RNA level; however, RNase J1 depletion in the absence of RNase J2 resulted in an increase in total mRNA half-life from 2.6 min to 3.6 min [45]. This increase is smaller in comparison to what was observed for RNase E depletion in* E. coli* but comparable to what was observed for PNPase deletion in* B. subtilis* (see the following). Thirty percent of total transcripts are targeted by RNase J1 revealing a wide action of this RNase [13]. In* B. subtilis*, the paradigm for RNA decay is an endonucleolytic cleavage by RNase Y, giving access to RNases J for 5′ to 3′ exonucleolytic activity, whereas PNPase performs a 3′ to 5′ exonucleolytic activity [13]. As studies were performed on depleted strains but not on a null-mutant, the global role of RNase Js could be underestimated as a residual RNase J activity may still be present. A recent study showed that it is possible to inactivate both* rnjA* and* rnjB* genes in* B. subtilis* [49]. The* rnjA* mutant is viable with a long doubling time (76 min instead of 26 min) with defects in sporulation, competence, and cell morphology, while the* rnjB* mutant has a growth rate similar to that of the wild-type strain [50].

Results from a saturated transposon mutagenesis suggested that* rnjA* and* rnjB* genes were essential in* S. aureus* [51]. However, Linder and colleagues succeeded in deleting both* rnjA* and* rnjB*. Each mutant exhibits poor growth at 42°C [52]. The fact that transposon mutagenesis is carried out at 42°C explains the discrepancy between the two studies. The heterodimer RNase J1/J2 exhibited highest catalytic efficiency. Inactivation of the RNase J2 active site by site-directed mutagenesis did not affect cell growth rate. This finding may indicate that RNase J2 is needed for RNase J1 efficiency but have a minor role in RNA processing [52]. Overexpression of RNase J1 can partially compensate the lack of RNase J2, suggesting that a homodimer RNase J1/J1 could be used in the absence of RNase J2. More experiments are needed to explain this compensation. A methodology for sequencing 5′ RNA ends was developed to decipher the impact of deleting RNase J1 or J2 [52]. Specific mRNAs are enriched in RNase J mutants, and clear mapping of the 5′ mRNA ends has led to the identification of RNase J roles in RNA processing. RNase J is involved in 16S rRNA precursor maturation. It processes 16S rRNA after endonucleolytic cleavage by RNase III as observed in* Sinorhizobium meliloti* and* B. subtilis* [44, 53]. In* S. aureus*, the maturation of* acpP* (acyl-carrier protein) mRNA exemplifies the role of RNase J1 and J2. In the absence of RNase J1, this essential mRNA is cleaved by RNase J2, thus demonstrating the activity of this enzyme in* S. aureus*.

RNase J is a complex enzyme made of two partners (J1 and J2) forming heteromers (either dimers or possibly tetramers)* in vivo*. RNase J2 has a narrower role than RNase J1; some functions of RNase J2 were observed in the RNase J1 mutant (e.g.,* acp* mRNA processing). However, the precise role of RNase J2 is not perfectly understood yet.

### 2.4. PNPase

The polynucleotide phosphorylase (PNPase) is a 3′ to 5′ exonuclease with a peculiar mechanism for RNA decay; whereas “classical” RNases cleave RNA molecules by hydrolysis, the PNPase cleaves RNAs by phosphorolysis involving an inorganic phosphate and releasing a nucleotide diphosphate. This contributes to the dual role of PNPase which also acts as a polymerase when the concentration of inorganic phosphate is lower than those of nucleotide diphosphate [54].

*S. aureus* encodes three 3′ to 5′ exonuclease orthologs, PNPase, RNase R, and YhaM. The global role of PNPase in RNA decay was determined at a genome scale, comparing wild-type and Δ*pnpA* isogenic strains [55]. While in the wild-type strain about 51% of total transcripts were degraded after five minutes, the percentage drops to 17% in the Δ*pnpA* strain [55]. PNPase depletion leads to cold shock sensitivity in* S. aureus*. PNPase may be important for the degradation of the large amount of* csp* (cold shock protein) transcripts after a cold shock induction as demonstrated in* E. coli* [56]. A recent study showed that* S. aureus* PNPase also interacts with RNase Y to degrade transcripts [40]. As an example,* agr* expression is decreased by the absence of RNase Y and this effect is suppressed in a* pnp* mutant. This phenotype is reversed when PNPase is expressed from an ectopic gene suggesting a direct role of PNPase in this process [40]. This study further demonstrates that RNA regulation implies a network involving several RNases.

## 3. The Multiprotein Degradation Complex: RNA Degradosome

The RNA degradosome was discovered during purification of RNase E from* E. coli* by two independent teams [58, 59]. Copurification of RNase E with PNPase provided a clue for the existence of a multiprotein complex involved in mRNA degradation. In* E. coli*, the major components of this complex are (i) RNase E, (ii) PNPase, (iii) RNA helicase B (RhlB), and (iv) the glycolytic enzyme enolase [60]. The function of these different partners was studied in* E. coli*. Thus, RNase E is an endonuclease sensing 5′ monophosphate ends [61] and PNPase is a 3′ to 5′ exonuclease (see the above). Interestingly, RhlB is a DEAD box helicase belonging to a ubiquitous protein family often possessing RNA-dependent ATPase activity [62]. RhlB unwinds RNA structures that can block PNPase action, as exemplified by the effect of the repetitive intergenic region (REP) on PNPase [63]. This role has been further supported by the demonstration that the RNA degradosome requires ATP hydrolysis to degrade RNA containing REP [64]. The role of the last partner enolase, a glycolytic enzyme, remains enigmatic. As the enolase belongs to glycolytic metabolism, it might sense (i) the energetic state of the cell or (ii) phosphosugar stress [65].

The existence of an RNA degradosome in* B. subtilis* was proposed with RNase Y as the central partner instead of RNase E. Protein-protein interactions demonstrated by a bacterial two-hybrid system led to the identification of RNases J1/J2, PNPase, a RNA helicase CshA, enolase, and phosphofructokinase, another glycolytic enzyme, as RNase Y partners [33]. Phosphofructokinase interacts with major partners of the degradosome (PNPase, RNase Y, and the RNases J1, J2) but also with RNase III which has not been identified as part of the degradosome.

A similar approach was used to identify the* S. aureus* degradosome [66] and led to the identification of the same partners, that is, enolase (SAR0832), phosphofructokinase (SAR1777), a DEAD box RNA helicase (SAR2168), PNPase (SAR1250), RNase J1 (SAR1063), RNase J2 (SAR1251), and RNase Y (SAR1262), with an additional partner, the RNase RnpA (see the following). RnpA interacts only with CshA interacting itself with enolase, phosphofructokinase and RNase Y.* S. aureus* and* B. subtilis* degradosome components are similar; however, the interactions between the different partners seem to be simpler in* S. aureus* [66]. In* B. subtilis*, each partner interacts with at least three other partners whereas, in* S. aureus*, each partner seems to interact with two only other partners.

## 4. Other RNases 

Until now, few RNases have been studied directly in* S. aureus*, and putative roles for the others are predicted based on assignments from other organisms. Among them, RNase P, a nucleoprotein complex shared by all kingdoms of life, removes 5′ extra-nucleotides from tRNA precursors [67]. Where known in bacteria, it is composed of a ribozyme (M1 RNA alias RnpB), RNA possessing catalytic activity, and a protein (protein C5 alias RnpA) expressed from the* rnpB* and* rnpA* genes, respectively [68]. Besides its impact on the maturation of tRNA 5′ ends, RNase P is involved in the maturation of 4.5S RNA precursor, polycistronic mRNA of histidine operon, tmRNA and some RNA phages [69]. A paralog of the RnpA moiety of RNaseP was identified in* S. aureus* sharing only 24% amino acid identity with* E. coli* RnpA; all conserved amino acids proved to be essential (Table 1). Interestingly, a recent study searching for new antimicrobial compounds led to a compound interacting with RnpA, suggesting that essential RNases might be effective drug targets [70].

Other RNases such as the 3′ to 5′ exonuclease RNase R that processes 3′ tRNA ends [71] and the endonuclease RNase Z that removes the 3′ tRNA termini [72] are conserved in* S. aureus*. Potential non-tRNA targets of RNase Z have been searched in* E. coli* by microarrays; the amount of more than 150 mRNAs had been increased in the* rnz* mutant as compared to the wild-type isogenic strain, possibly indicating a role of RNase Z in processing of a more wide range of RNAs than just tRNAs or indirect effect. However, so far, nothing is known concerning the role of RNase Z in* S. aureus*.

The 5S rRNA precursor in bacteria with low GC content is matured by the specific RNase M5 [73, 74]. The ribosomal protein L18 is proposed to alter precursor conformation, stimulating 5S rRNA processing, whereas the ribosomal protein L5 inhibits cleavage [75]. An RNase M5 ortholog sharing 53% amino acid identity is present in* S. aureus*. However as for RNase Z and R, it has not been studied in* S. aureus* and its impact remains to be established.

Members of the RNase H family cleave RNAs in an RNA/DNA duplex [76]. These enzymes perform diverse fundamental cellular processes, including DNA recombination, replication and repair, and RNA interference [77]. The family is divided in three subclasses, HI to HIII [78], which are expressed in* B. subtilis* from paralog genes* rnhA*,* rnhB*, and* rnhC*, respectively. In* B. subtilis*, only RNases HII and HIII possess RNase H activity [76] and are essential. Even if crystallographic structure of RNase HIII was obtained by diffraction [79], the* rnhA*,* rnhB*, and* rnhC*, genes are also present in* S. aureus* and await characterization.

A 3′ to 5′ exonuclease degrading single strand RNAs, encoded by the* yhaM* gene, was purified from a* B. subtilis* strain lacking PNPase and RNase R [80]. The* yhaM* deletion alone did not affect growth in the tested conditions nor the bulk mRNA half-life; however, strains lacking YhaM and either RNase R or PNPase were unable to grow at low temperature [80]. The* S. aureus yhaM* ortholog gene expresses Cbf1 which was initially shown to be a DNA-binding protein involved in plasmid replication [81]. Purified Cbf1 has RNase activity, but to date, its role at a genomic level was not well understood.

In* E. coli*, degradation of short oligonucleotides is performed by the essential oligoribonuclease Orn [82].* B. subtilis* lacks an* orn* ortholog, but the corresponding activity is performed by two paralogs named nanoRNase A and nanoRNase B (encoded by* nrnA* and* nrnB*, resp.), which act together to scavenge and recycle nucleotides for new RNA transcripts [83]. Genome sequence analyses indicate the existence of an* nrnA* orthologous gene in* S. aureus*, while to date, no study concerning this RNase family has been performed.

## 5. Acquired Ribonucleases

RNases mainly belong to species core genomes. For instance, RNase III, RNase J1, RNase J2, and RNase Y are found in all isolates of the* S. aureus* species. However, several acquired RNases have been described. These enzymes are, so far, part of toxin/antitoxin (TA) systems. TA systems can be divided in five groups according to the antitoxin function [84]. The two main TA systems are type I TA, in which the antitoxin is a small antisense RNA that base-pairs with toxin mRNA, and type II TA, in which the antitoxin is a protein acting on a posttranslational step [84]. Several toxins or antitoxins exhibit RNase properties as is the case for the well characterized TA system MazE/MazF [85]. The MazF ribonuclease recognizes a specific sequence that may vary between species [85]. In* E. coli*, MazF recognizes the 5′ end of ACA and cleaves just before the cytosine (A∧CA with “∧” represents the cleavage site), whereas in* S. aureus*, SaMazF cleaves inside a five-base sequence U∧ACAU [86, 87]. Up to now, three TA systems exhibiting RNase activity were described in* S. aureus*, SaMazE/F, SaPemI/K, and YefM-YoeB [88–91].

In* E. coli*, expression of MazF causes global mRNA degradation leading to reprogramming and growth arrest; cell death is rescued by MazE [92]. However, MazE cannot rescue cells in the presence of a quorum-sensing-induced pentapeptide that competes with MazE and thus cell death is induced [92]. The quorum-sensing allows communication between bacteria and this pentapeptide acts as a death inductor. In* E. coli*, MazF is involved in the cleavage of (i) mRNAs at ACA sequences in the vicinity of the AUG start codon and (ii) 16S rRNA within the 30S subunit [93]. Modified ribosomes are required for translation initiation of these leaderless mRNAs, which are likely involved in stress adaptation [93]. In* S. aureus*, MazF cleaves at U∧ACAU which is a relatively abundant sequence, for instance, inside the* sraP* gene, coding for a protein involved in the cell adhesion and thus virulence [87].

Recently, another role of ribonuclease-encoding TA system has been described for SaPemI/K [88]. This plasmid-encoded TA system, in addition to its role in plasmid maintenance, seems to play a global regulatory role in virulence by altering the translation of a large pool of genes [88].

The last system, YefM-YoeB, has a ribosome-dependent RNase activity. The toxin binds the A site of the 50S ribosomal subunit and then cleaves the mRNA three base pairs after the start codon [91]. In addition, SaYoeB exhibits a ribosome-independent RNase activity* in vitro* by cleaving free mRNA consistent with that previously observed in* E. coli* [91].

RNases encoded by TA systems may have a global impact on staphylococcal posttranscriptional regulation. Global scale experiments of these systems need to be performed.

## 6. Non-RNase Partners of RNA Decay: RppH and Hfq

**Figure 3:**
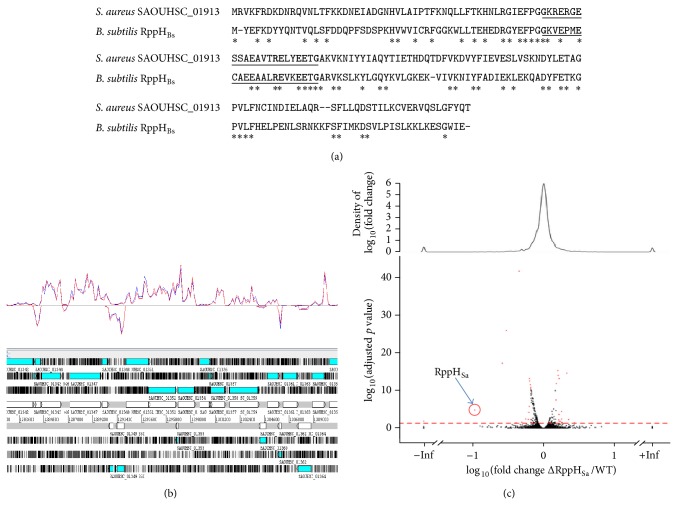
SAOUHSC_01913, a putative pyrophosphohydrolase in* S. aureus*. (a) Protein sequence alignment of RppH_Bs_ from* B. subtilis* AG1839 (Genbank accession number CP008698.1) and SAOUHSC_01913 (RppH_Sa_) from* S. aureus* NCTC8325 (Genbank accession number NC_007795). The NUDIX motif is underlined and the conserved amino acid residues within the NUDIX region are in bold. Identical residues between both proteins are indicated by a star. (b) Artemis visualization of a randomly chosen region within the chromosome of HG003. The bottom part represents the six open reading frames, indicated by blue arrows. The upper part represents strand coverage of HG003 in red and HG003 Δ*rppH* in blue. No coverage difference is observed between the two strains. (c) Volcano plot representation (a scatter-plot constructed by plotting the negative log of the *p* value on the *y*-axis and the log of the fold change between the two conditions on the *x*-axis) of DEseq analysis between HG003 and HG003 Δ*rppH*. The red dots correspond to mRNAs differentially expressed according to the fold change and the adjusted *p* value (fold change of 1.5 and a *p* value > 0.05). The most differentially expressed RNA corresponds to* rppH* mRNA itself as indicated by the red circle.

Enzymes without RNase activities, such as RppH and Hfq, can be involved in the decay of bulk RNA. RppH, for RNA pyrophosphate hydrolase, triggers RNA degradation by removing the 5′ pyrophosphate of mRNA [94]. The remaining 5′ monophosphate RNAs are then more efficiently targeted by RNase E. RppH belongs to the NUDIX (Nucleoside Diphosphate linked to X) protein family, which exhibits phosphohydrolase activity [95]. In* E. coli*, RppH is responsible for the acceleration of the decay of hundreds of transcripts, demonstrating its importance in RNA stability [94]. The purified* E. coli* RppH protein did not present any substrate specificity, at least in terms of the 5′-end nucleotide [94]. However, unexpected substrate specificity was recently reported for RppH_Bs_, the* B. subtilis* RppH ortholog [96, 97]. RppH_Bs_ drives pyrophosphate hydrolysis of a synthetic RNA when (i) at least two and preferably three or more nucleotides are unpaired at the 5′ end and (ii) if the second nucleotide is a guanosine and the third nucleotide is preferentially a purine [96]. This observation has been further explained by a RppH_Bs_ crystallographic study revealing a binding pocket that fits a guanosine in the second position of substrates [97]. Yet the crystal structure of* Bdellovibrio bacteriovorus* RppH leads to a different interpretation, RppH recognizes the first nucleotide of the sequence. These results prompted us to inactivate and identify possible substrate specificity in* S. aureus* (Bonnin and Bouloc, unpublished data). Based on protein sequence identity (38% amino acid identity with RppH_Bs_) and conserved synteny, we identified SAOUHSC_01913 as the gene expressing the* S aureus* RppH ortholog (RppH_Sa_). The* rppH* gene of* S. aureus* strain HG003 [98] was deleted as described [99]. Total RNAs of* S. aureus* HG003 and HG003 Δ*rppH* in exponential phase were extracted, sequenced by RNA-seq and transcriptomes of these strains were compared using DeSeq tools [100]. Unexpectedly, very few differences were observed between the two transcriptomes with only four transcripts stabilized in the* rppH* mutant (Figure 3). None of them had a guanosine in the second position and they did not share any apparent common features. These results indicate a minor role of RppH_Sa_ in the tested condition possibly due to the presence of a second RppH-like enzyme that could compensate for the absence of RppH_Sa_ (SAOUHSC_01913).

A key non-RNase player in RNA processing and decay is the RNA-binding protein Hfq. Discovered more than forty years ago in* E. coli*, Hfq was identified as an essential host factor for bacteriophage Q*β* [101]. In many bacteria, Hfq promotes activity of regulatory RNAs by protecting them against degradation and stimulating pairing with their targets. Consequently, sRNA-regulated genes can be both posttranscriptionally up- or downregulated and the absence of Hfq can generate numerous phenotypes; for an in-depth review, see [102]. While the first solved Hfq crystal structure was that of* S. aureus* (Hfq_Sa_) [103], its function in* S. aureus* remains unknown. The *hfq*_Sa_ deletion showed no phenotype when tested on over 1,500 tested growth conditions [99]. In contrast to the multiple Hfq phenotypes reported for enteric bacteria, the absence of Hfq in* S. aureus* as well as in* B. subtilis* has no impact on sRNA-mediated regulation, reviewed in [21]. It is conceivable that *hfq*_Sa_ is poorly or not expressed in the studied strains. In one report, *hfq*_Sa_ deletion in strains where Hfq was detected resulted in decreased toxicity and virulence, and over a hundred genes showed differential expression in an* hfq*-mutant compared to a wild-type strain using microarrays [104]. The observed discrepancy between the different studies may lie in the fact that the strains used for these experiments are different in some aspects even if they belong to the same lineage, that is,* S. aureus* NCTC8325. Surprisingly, the *hfq*_Sa_ gene fails to substitute for the* hfq* of* Salmonella* in sRNA-mediated regulation [105]. Altogether, these results suggest that Hfq_Sa_ does not play a central role in posttranscriptional regulation. Further investigation will be necessary to understand the exact role of Hfq in* S. aureus*.

## 7. Concluding Remarks

Recent studies on the RNase functions in* S. aureus* indicate that the scheme for RNA decay is similar to that in the low G+C content Firmicutes model* B. subtilis*. For instance, the absence of RNase E, replaced by RNase Js and Y, is also observed in* S. aureus*. RNases are key players of posttranscriptional regulation and therefore are involved in virulence factor regulation. As an example, RNase III controls the expression of factors involved in cell adhesions or factor involved in immunity escape* via* the degradation of sRNA/mRNA duplexes.

Up to now, the impacts of only three RNases, that is, RNase III, Y, and Js, have been studied on the genome scale. Further studies will be needed to elucidate the precise roles of the other RNases present in* S. aureus* and their potential effects on virulence gene regulation.

## Figures and Tables

**Table 1:** Ribonucleases in *S. aureus*.

Ribonuclease	Gene	Function^a^	Amino acid identity between NCTC8325 and *B. subtilis* 168 orthologs^c^	Amino acid identity between NCTC8325 and *E. coli* MG1655 orthologs^c^	Nomenclature N315	Nomenclature NCTC8325	Essentiality^b^
RNase III	*rnc *	ds-RNA endonuclease∗	0.49	0.34	SA1076	SAOUHSC_01203	N

Mini-III	*mrnC *	ds-RNA endonuclease^¤^	0.56	None	SA0489	SAOUHSC_00512	N^b^

RNase Y	*rny*/*cvfA *	ss-RNA endonuclease∗	0.69	None	SA1129	SAOUHSC_01263	N

RNase J1	*rnjA *	Strong 5′-3′ exonuclease activity∗ ss-RNA endonuclease	0.67	None	SA0940	SAOUHSC_01035	N∗∗

RNase J2	*rnjB *	Weak 5′-3′ exonuclease activity∗ ss-RNA endonuclease?	0.50	None	SA1118	SAOUHSC_01252	N∗∗

RNase P	*rnpA *	Endonucleolytic cleavage of RNA, removing 5′-extranucleotides from tRNA precursor with *rnpB* ribozyme∗	0.49	0.24	SA2502	SAOUHSC_03054	Y

RNase Z	*Rnz *	Endonucleolytic cleavage of RNA involved in removing extra 3′ nucleotides from the tRNA precursor^¤^	0.45	0.41	SA1335	SAOUHSC_01598	Y

RNase M5	*rnmV *	ds-RNA endonuclease, maturation of 5S rRNA^¤^	0.53	None	SA0450	SAOUHSC_00463	N

PNPase	*pnpA *	3′-5′ Exonuclease∗	0.68	0.50	SA1117	SAOUHSC_01251	N

RNase R	*Rnr *	3′-5′ Exonuclease^¤^	0.55	0.37	SA0735	SAOUHSC_00803	Y

YhaM	*yhaM *	3′-5′ Exonuclease^¤^	0.52	None	SA1660	SAOUHSC_01973	N

RNase HI	*ypqD/rnhA *	RNase HI-family protein of unknown function^¤^	0.33	None	SA1266	SAOUHSC_01443	N

RNase HII	*rnhB *	Endonuclease, degradation of RNA/DNA duplexes^¤^	0.47	0.44	SA1087	SAOUHSC_01215	N

RNase HIII	*rnhC *	Endonuclease, degradation of RNA/DNA duplexes^¤^	0.46	None	SA0987	SAOUHSC_01095	N

NanoRNase A	*nrnA *	Oligoribonuclease, 3′,5′-bisphosphate nucleotidase^¤^	0.49	None	SA1526	SAOUHSC_01812	N

^a^Function: ∗demonstrated experimentally; ^¤^function based on results of *B. subtilis* or *E. coli* studies.

^b^Essentiality: Y demonstrated experimentally using transposon mutagenesis [51]; N not essential demonstrated experimentally, N^b^ not essential based on *B. subtilis* studies. ∗∗RNase J1 and J2 are essential at 42°C but not at lower temperatures [51, 52].

^c^Accession numbers: *B. subtilis *168, NC_000964.3; *E. coli* MG1655; NC_000913.3.
